# Supplementary material for: Solution-processed nanographene distributed feedback lasers
Source: Nat Commun. 2019 Jul 25;10:3327. doi: 10.1038/s41467-019-11336-0 (PMC6658550; doi:10.1038/s41467-019-11336-0)
Supplement: Supplementary file 1 — Supplementary Information [file 41467_2019_11336_MOESM1_ESM.pdf]

Supplementary Information

Solution-processed nanographene distributed feedback lasers

Víctor Bonal[a], Rafael Muñoz-Mármol[a], Fernando Gordillo Gámez[b], Marta Morales-Vidal[a], José M. Villalvilla[a], Pedro G. Boj[c], José A. Quintana[c], Yanwei Gu[d], Jishan Wu[d], Juan Casado[b], María A. Díaz-García[a]

[a]*Departamento Física Aplicada and Instituto Universitario de Materiales de Alicante, Universidad de Alicante, Alicante 03080, Spain*

[b]*Department of Physical Chemistry, University of Málaga, Andalucía Tech., Campus de Teatinos s/n, Málaga 29071, Spain*

[c]*Departamento Óptica, Farmacología y Anatomía; and Instituto Universitario de Materiales de Alicante, Universidad de Alicante, Alicante 03080, Spain*

[d]*Department of Chemistry, National University of Singapore, 3 Science Drive 3, 117543, Singapore*

Correspondence and requests for materials should be addressed to: Prof. María A. Díaz-García (e-mail: maria.diaz@ua.es), for laser data; Dr. Juan Casado (e-mail: casado@uma.es), for low-temperature pholuminescence and Raman data; and Prof. Jishan Wu (e-mail: chmwuj@nus.edu.sg), for nanographene synthesis.

(a)

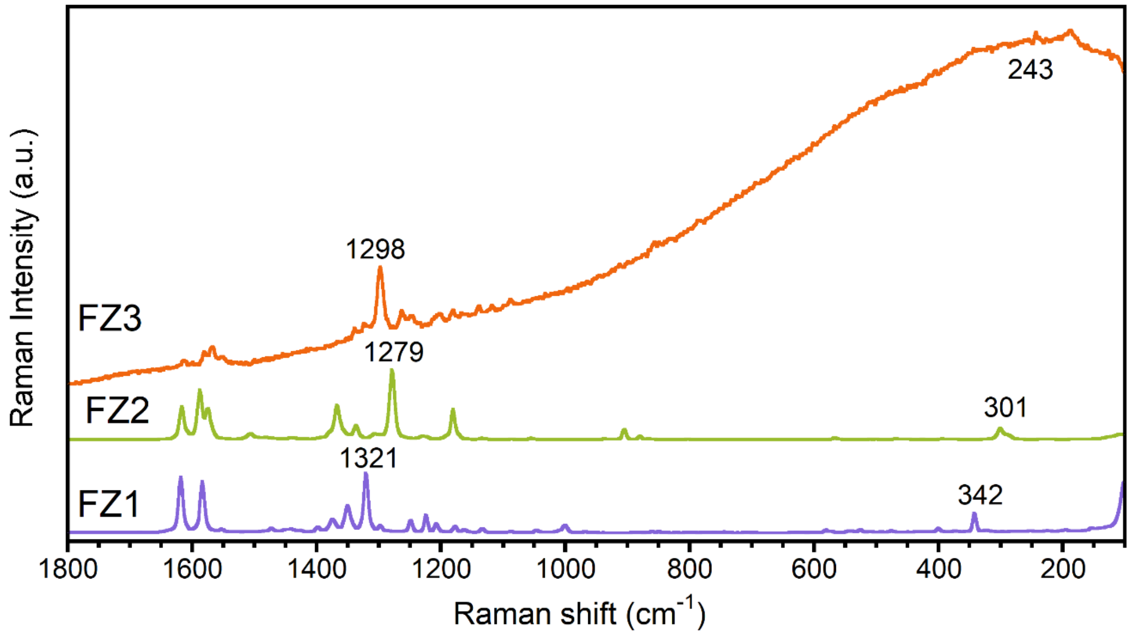

(b)

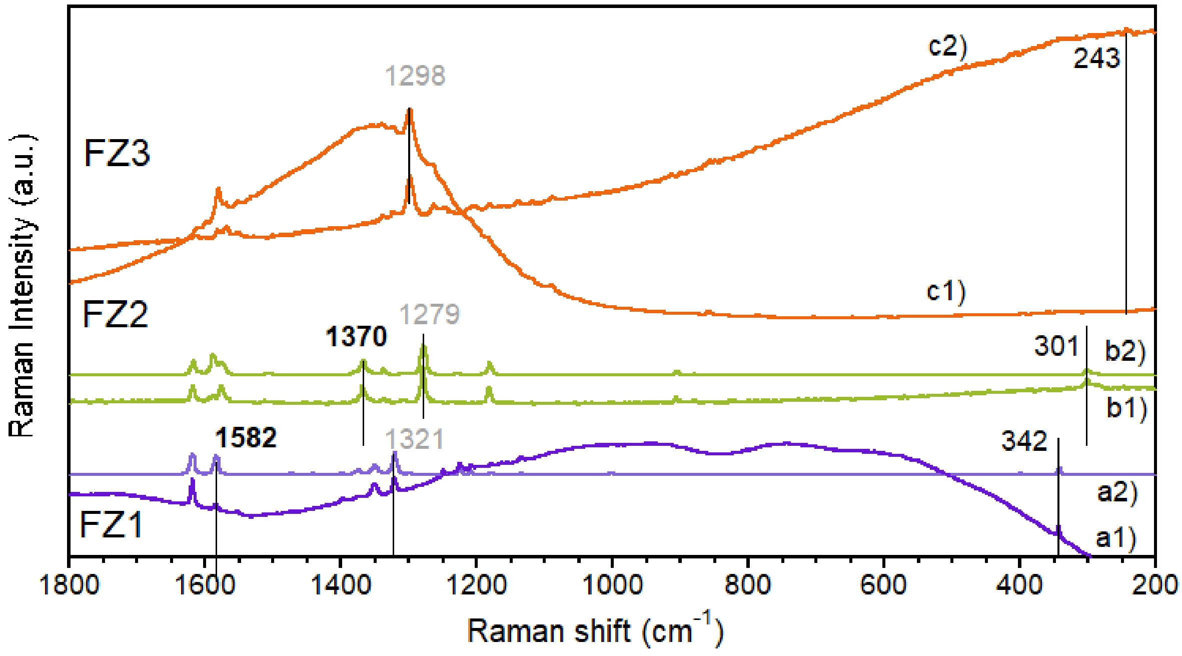

Supplementary Figure 1. **Raman spectra of the three nanographenes in solid state at room temperature.** **(a)** Spectra in resonant conditions: The excitation wavelength, λ_{exc}, was 1064 nm, for FZ1 and FZ2, and 785 nm for FZ3; **(b)** Spectra in pre-resonant Raman conditions: curves a1 (λ_{exc} = 532 nm), b1 (λ_{exc} = 633 nm); and c1 (λ_{exc} = 1064

nm), for FZ1, FZ2 and FZ3 respectively. For comparison, the same curves shown in Figure a) are also shown here. Source data are provided as a Source Data file in the Institutional Repository of the University of Alicante [http://hdl.handle.net/10045/92007].

By comparing the spectra of one given compound, it is seen that they are essentially identical for different laser excitation lines. It is a well-known feature of the Raman spectra of poly-conjugated (coloured) compounds that they are rather independent of the excitation laser wavelength used in the Raman experiment (when this is in the visible/NIR range). This is because the Raman intensity is dictated by the vibronic term associated to the main electronic absorption band, which is that dominating the whole electronic absorption spectrum (in visible/NIR region) of these molecules. The similarity of the Raman spectra for the same FZ*n* compound taken with different excitation lines is a corroboration of discussion of the dominance of the vibronic mechanism in the Raman spectra.

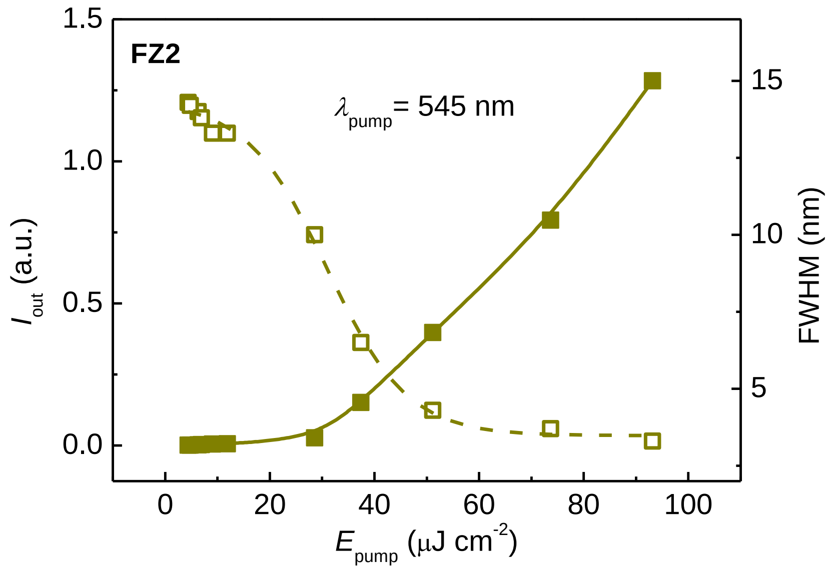

Supplementary Figure 2. Amplified spontaneous emission (ASE) plots used for ASE threshold determination. The numerical value of the ASE threshold for a given film was determined from the plot of its emission linewidth (defined as the full with at half of maximum intensity, FWHM) versus pump energy (E_{pump}), as the E_{pump} value at which FWHM decays to half of its maximum value. Such a plot for a 1 wt% FZ2-doped PS film is represented by open squares in the right axis of the figure. The full line is a guide to the eye. ASE threshold determination from the plot of the output intensity (I_{out}) at the wavelength at which ASE appears versus E_{pump} (such a plot for the 1-wt% FZ2-doped PS film is represented by full squares in the left axis), as the E_{pump} value at which a drastic slope change occurs (method used for DFB threshold determination) would involve larger errors, since the change in slope is gradual and hence not well-defined. Source data are provided as a Source Data file in the Institutional Repository of the University of Alicante [http://hdl.handle.net/10045/92007].

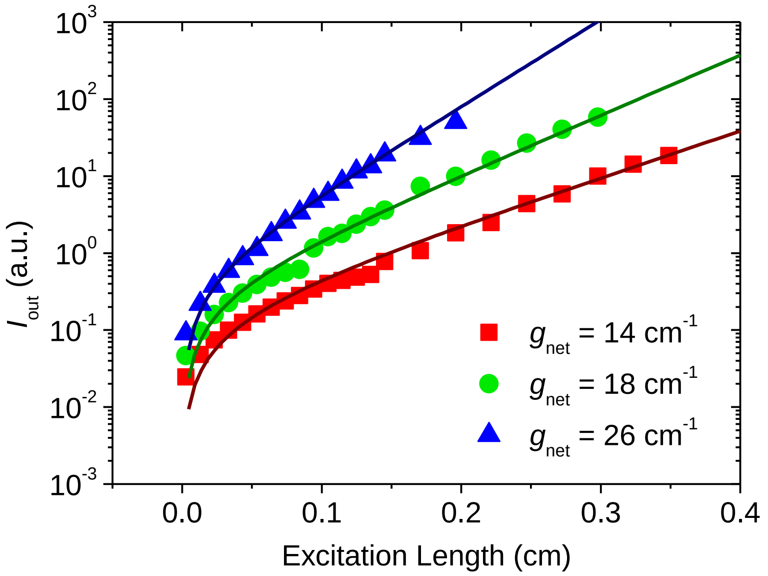

Supplementary Figure 3. Amplified spontaneous emission (ASE) Variable Length Stripe study for net gain coefficients determination. Emission intensity at the wavelength at which ASE appears (λ = 590.5 nm) versus the length of the excitation stripe for a 1 wt% FZ2-doped polystyrene film at pump energy densities of 140, 270 and 520 μJ cm^{-2} (squares, circles and triangles, respectively). The solid lines are fits to the data using Equation 2 (see experimental methods in manuscript), from which net gain coefficients, g_{net}, were obtained (values indicated in the figure). Source data are provided as a Source Data file in the Institutional Repository of the University of Alicante [http://hdl.handle.net/10045/92007].

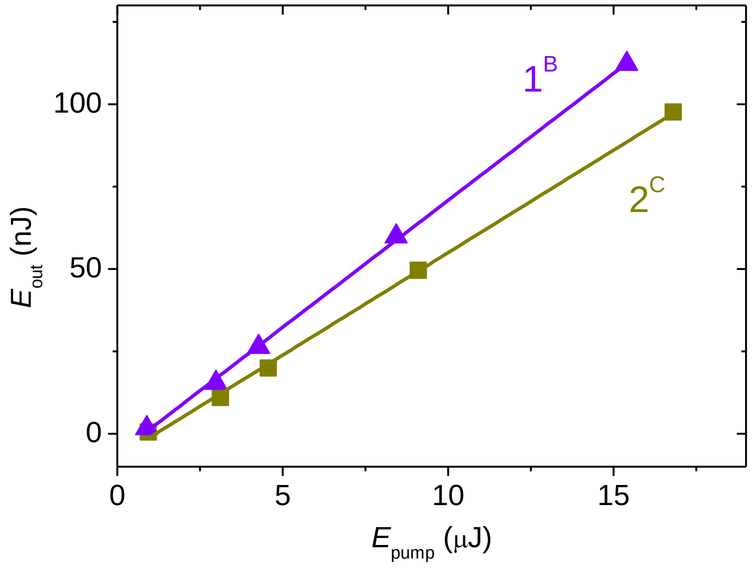

Supplementary Figure 4. Laser slope efficiency, LSE, measurements. Plots of total output pulse energy versus pump pulse energy (above threshold) for laser devices 1^B and 2^C, based on FZ1 and FZ2 respectively (parameters in Table 2 in manuscript). From linear fits to data, LSE values (error ≈10%) of 0.77% (1^B) and 0.62% (2^C) were obtained. Source data are provided as a Source Data file in the Institutional Repository of the University of Alicante [http://hdl.handle.net/10045/92007].
