# Supplementary material for: Solution-processed nanographene distributed feedback lasers
Source: Nat Commun. 2019 Jul 25;10:3327. doi: 10.1038/s41467-019-11336-0 (PMC6658550; doi:10.1038/s41467-019-11336-0)
Supplement: Supplementary file 3 — Reporting Summary [file 41467_2019_11336_MOESM3_ESM.pdf]

Corresponding author(s): María A. Díaz-García

# Lasing Reporting Summary

Nature Research wishes to improve the reproducibility of the work that we publish. This form is intended for publication with all accepted papers reporting claims of lasing and provides structure for consistency and transparency in reporting. Some list items might not apply to an individual manuscript, but all fields must be completed for clarity.

For further information on Nature Research policies, including our data availability policy, see Authors & Referees.

## Experimental design

**Please check: are the following details reported in the manuscript?**

1. Threshold

Plots of device output power versus pump power over a wide range of values indicating a clear threshold

☒ Yes ☐ No

They are shown as log-log plots (following the suggestion of Reviewer 3) in Fig. 3b.

2. Linewidth narrowing

Plots of spectral power density for the emission at pump powers below, around, and above the lasing threshold, indicating a clear linewidth narrowing at threshold

☒ Yes ☐ No

Plots of the emission linewidth versus pump energy for amplified spontaneous emission (ASE) are shown in Supplementary Fig. 2. Similar plots for lasing are shown in Fig. 3a.

Resolution of the spectrometer used to make spectral measurements

☒ Yes ☐ No

The resolution of the spectrometers used is indicated in the Methods section, page 19 and 20.

3. Coherent emission

Measurements of the coherence and/or polarization of the emission

☒ Yes ☐ No

Information about the polarization of the ASE emission in page 6 and of the polarization of the emitted laser light in page 13.

4. Beam spatial profile

Image and/or measurement of the spatial shape and profile of the emission, showing a well-defined beam above threshold

☒ Yes ☐ No

Beam spatial profile information provided by images of the emitted light shown in Fig. 2d. Also, spectral beam profile information illustrated in Fig. 2c.

5. Operating conditions

Description of the laser and pumping conditions
*Continuous-wave, pulsed, temperature of operation*

☒ Yes ☐ No

The pumping conditions are described in the section Methods-Optical experiments (pages 19 and 20). Values of the pump wavelength and its corresponding pulsewidth for each case shown in Tables 1 and 2.

Threshold values provided as density values (e.g. W $cm^{-2}$ or J $cm^{-2}$) taking into account the area of the device

☒ Yes ☐ No

Laser threshold values are shown in both types of units in Table 2. The area of the excitation area is indicated in the section Methods-Optical experiments.

6. Alternative explanations

Reasoning as to why alternative explanations have been ruled out as responsible for the emission characteristics
*e.g. amplified spontaneous, directional scattering; modification of fluorescence spectrum by the cavity*

☒ Yes ☐ No

Besides a laser characterization, we have performed amplified spontaneous emission (ASE) study in films without laser resonators.

7. Theoretical analysis

Theoretical analysis that ensures that the experimental values measured are realistic and reasonable
*e.g. laser threshold, linewidth, cavity gain-loss, efficiency*

☒ Yes ☐ No

The experimental laser wavelengths comply very well with the Bragg condition. Thanks to the fine control of the fabrication method to change the grating period and the uniformity of the film thickess, we are able to design with preccision the emission wavelength of the devices. This is discussed in pages 11 and 12.

8. Statistics

Number of devices fabricated and tested

☒ Yes ☐ No

Commented through the last sentence of the Methods section and through a statement in the caption of Fig. 1d to account for the error bars shown.

Statistical analysis of the device performance and lifetime (time to failure)

☒ Yes ☐ No

Operational lifetime analysis in Fig. 3d and data extracted from it in Table 2. Discussion in Pages 14 and 15. In addition we have commented on the lifetime and also the stability of the ASE emission in Page 10.



March 2018
